# Severe growth hormone deficiency and pituitary malformation in a patient with chromosome 2p25 duplication and 2q37 deletion

**DOI:** 10.1186/1755-8166-7-41

**Published:** 2014-06-19

**Authors:** Annalisa Vetro, Sara Pagani, Margherita Silengo, Mariasavina Severino, Elena Bozzola, Cristina Meazza, Orsetta Zuffardi, Mauro Bozzola

**Affiliations:** 1 Biotechnology Research Laboratory, Fondazione IRCCS San Matteo, Pavia, Italy; 2 Department of Internal Medicine and Therapeutics, Pediatric and Adolescent Unit, University of Pavia, Fondazione IRCCS San Matteo, Pavia, Italy; 3 Department of Public Health and Paediatric Sciences, University of Torino, Torino, Italy; 4 Neuroradiology Unit, Istituto Giannina Gaslini, Genoa, Italy; 5 Department of Pediatric Medicine, IRCCS Ospedale Pediatrico Bambino Gesù, Rome, Italy; 6 Department of Molecular Medicine, University of Pavia, Pavia, Italy; 7 Internal Medicine and Therapeutics Department, University of Pavia, Fondazione IRCCS San Matteo, Piazzale Golgi 2, 27100 Pavia, Italy

**Keywords:** 2p duplication, 2q deletion, Growth hormone deficiency, Pituitary hypoplasia

## Abstract

We report on a male child ascertained at 4.8 years of age with severe growth failure, growth hormone (GH) deficiency, psychomotor delay with prevalent speech impairment, and a distinct phenotype. An evaluation of his hypothalamic-pituitary region by Magnetic Resonance Imaging (MRI) revealed pituitary hypoplasia with pituitary stalk interruption and ectopic posterior pituitary lobe, which are considered prognostic markers of permanent GH deficiency. Prenatal chromosome analysis because of increased nuchal translucency revealed a normal male karyotype, whereas postnatal high resolution banding raised the suspicion of a 2q abnormality. Subsequently, array Comparative Genomic Hybridization (array-CGH) revealed a *de novo* complex genomic rearrangement consisting of a 2p25 duplication and a 2q37 deletion: arr[hg19] 2p25.3p25.1(30,341-9,588,369)x3,2q37.2q37.3(235,744,424-243,041,305)x1. FISH analysis showed that the abnormal chromosome 2 mimicked the derivative of an inversion with the duplicated 2p region located distally at 2q. This is, to the best of our knowledge, the first case with distal 2p25 duplication and 2q37 deletion and pituitary malformation leading to GH deficiency.

## Background

The overall frequency of sub-microscopic causative deletions and duplications detected by chromosomal microarrays (CMA) in patients with intellectual disability/multiple congenital malformations, is around 15-20%, significantly higher in respect to that provided by conventional karyotyping [1].

Chromosome 2q37 deletion has been reported in more than 100 patients most of whom display short stature, obesity, brachydactily and intellectual deficiency, which are also observed in Albright syndrome [2]. The *HDAC4* gene has notably been established as responsible for brachymetaphalangy and intellectual deficit observed in these individuals [2,3]. Distal 2p duplication has also been reported although much more rarely and mostly in the context of inv dup 2p syndrome [4,5]. The clinical phenotype includes pre- and post-natal growth retardation, psychomotor delay, facial dysmorphism and, less frequently congenital heart malformations. To our knowledge only three subjects have been reported to have both the 2q deletion and 2p duplication two resulting de novo and one being the derivative of a maternal pericentric inversion [6,7]. Due to the complexity of the chromosomal rearrangement and its rarity, a clinical diagnosis of this condition is almost impossible. Here, we report on a four year-old child harboring a *de novo* 2p25 duplication and 2q37.1-qter deletion presenting with severe psychomotor delay and complete GH deficiency due to pituitary hypoplasia. The molecular diagnosis had been significantly delayed since prenatal cytogenetics analysis, performed to investigate increased fetal nuchal translucency, resulted in a normal male karyotype. As a consequence, the patient’s condition in his early infancy was attributed to a Mendelian syndrome.

## Case presentation

The propositus was the second child of healthy non-consanguineous Italian parents. His father and mother were 40 and 37 years old, respectively. Ultrasound scan at the 22^nd^ week of gestation revealed an increased fetal nuchal translucency, while the amniocentesis showed a normal 46,XY fetal karyotype. The patient was born at term (40 weeks) by spontaneous vaginal delivery. The birth weight was 3340 g (0.51 SD), birth length 50 cm (0.53 SD), and OFC 36 cm (0.50 SD). Apgar scores were 9 and 9 at 1 and 5 minutes, respectively. Family history was unremarkable. The father’s height was 165.8 cm and the mother’s height was 148.5 cm with a mid-parental height of 163.5 cm. His eight year-old sister was healthy.

**Figure 1:**
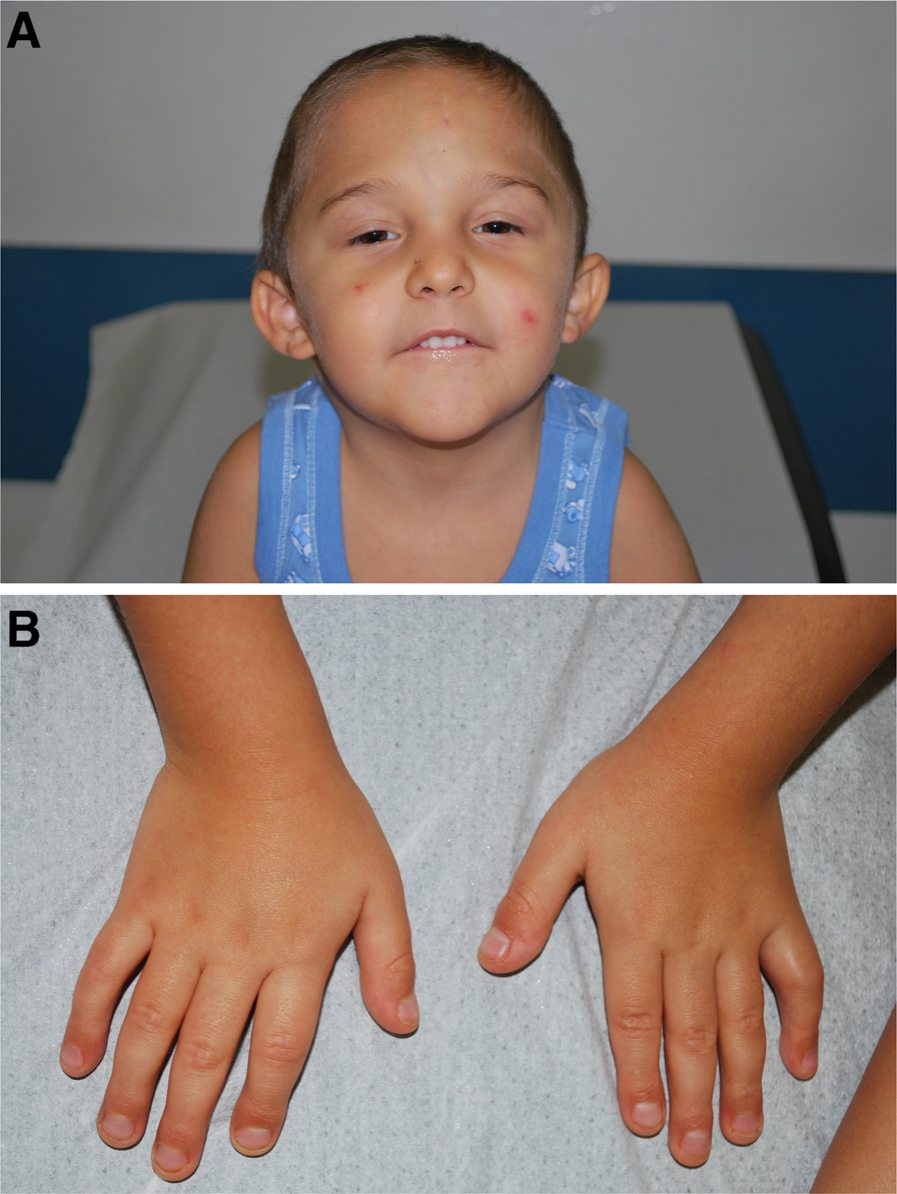
**Patient’s images. A)** Patient at of 7.5 years showing facial characteristics including right palpebral ptosis, low set ears, and small mouth with thin upper lip. **B)** Patient’s hands showing brachymetaphalangism.

**Figure 2:**
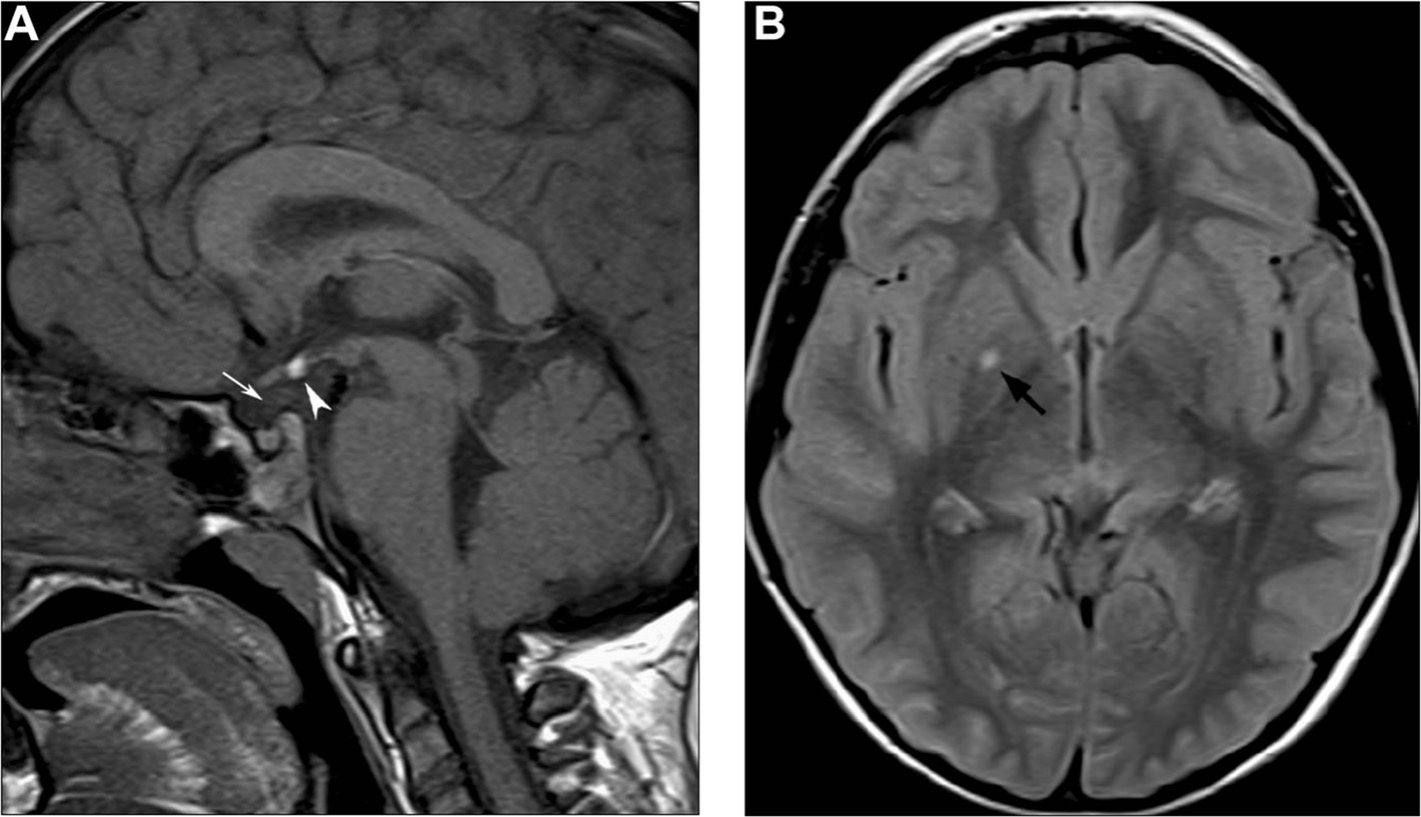
**Brain MRI of the patient at 4.8 years. A)** Unenhanced midline sagittal T1-weighted image shows hyperintensity corresponding to ectopic posterior pituitary lobe at the median eminence (arrowhead). The pituitary gland and sella turcica are small, and the pituitary stalk is not visible (white arrow). **B)** Axial FLAIR image reveals a small nonspecific hyperintense lesion in the right globus pallidus (black arrow).

**Figure 3:**
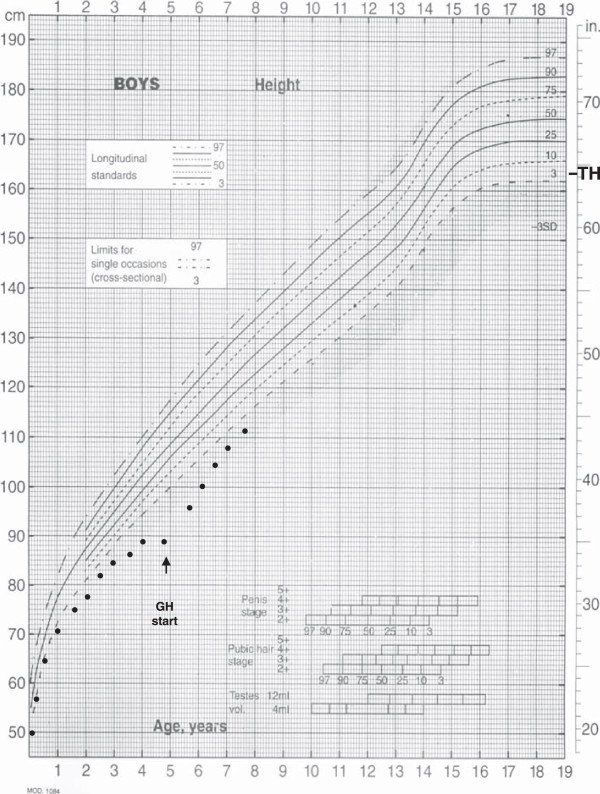
**Patient’s growth chart.** In the figure height measurements from birth to 7.5 years are reported.

At four months of age the parents noted the presence of axial hypotonia. When first seen at one year of age, he was noted to have dysmorphic features, including micro-brachycephaly due to premature closure of the anterior fontanelle, prominent forehead, up-slanting palpebral fissures, low set ears and sparse hair. Brain ultrasound was normal. All developmental milestones were delayed: he achieved head control at five months of age, sat unaided at nine months, and walked at 21 months. He was able to speak in complete sentences at four years. Because of a systolic murmur, at the age of three months a cardiac ultrasound examination revealed a mitral valve dysplasia with valve insufficiency, not requiring surgical repair. He underwent speech therapy sessions because of a mild perceptive hypoacusia found at four months.On physical examination at the age of 4.8 years his height was 89.1 cm (-4.05 SD), his weight was 11.9 kg (-0.57 SD), OFC was 48 cm (-1 SD) and growth velocity was reduced for age (4 cm/year, i.e. -2.1 SD). He presented a characteristic phenotype including slight trigonocephaly, right palpebral ptosis, low set ears, small mouth with a thin upper lip and micrognathia (Figure 1A), with small hands and feet with fleshy fingertips, brachymetaphalangism (Figure 1B), bilateral single palmar creases, short neck and scoliosis. Chronic diseases including intestinal malabsorption, celiac disease, constitutional skeletal diseases and hypothyroidism were excluded. Two pharmacological stimulus tests with arginine chlorohydrate and glucagon revealed a severe growth hormone (GH) deficiency (peak of 1.7 and 2 ng/ml, respectively), confirmed by IGF-I values: <25 ng/ml (-3.49 SD) that increased to 62.7 ng/ml after four subcutaneous GH injections suggesting good responsiveness to GH treatment (0.24 mg/Kg/week). No other pituitary hormone deficiencies were found to be associated. A brain MRI was performed with sagittal and coronal 3 mm-thick T1- and T2-weighted images for the hypothalamic-pituitary region, revealing pituitary hypoplasia, pituitary stalk interruption and an ectopic posterior pituitary lobe (Figure 2A). Additionally, a small focal nonspecific T2 hyperintensity was noted in the right globus pallidus that was unchanged at a 6-month brain MRI follow-up (Figure 2B). Substitutive GH therapy was started and led to a significant growth velocity increase as shown in Figure 3. In fact, after the first year of treatment, his height was 100 cm (-2.84 SD) and weight 15 Kg (-0.66 SD) with a significant increase in growth velocity to 8.5 cm/yr (+2.51 SD). After the second year of GH therapy, his height and weight were 107.9 cm (-2.31 SD) and 17.5 Kg (-0.77 SD) respectively, with a significant increase in his growth rate to 7.9 cm (+3.04 SD).

**Figure 4:**
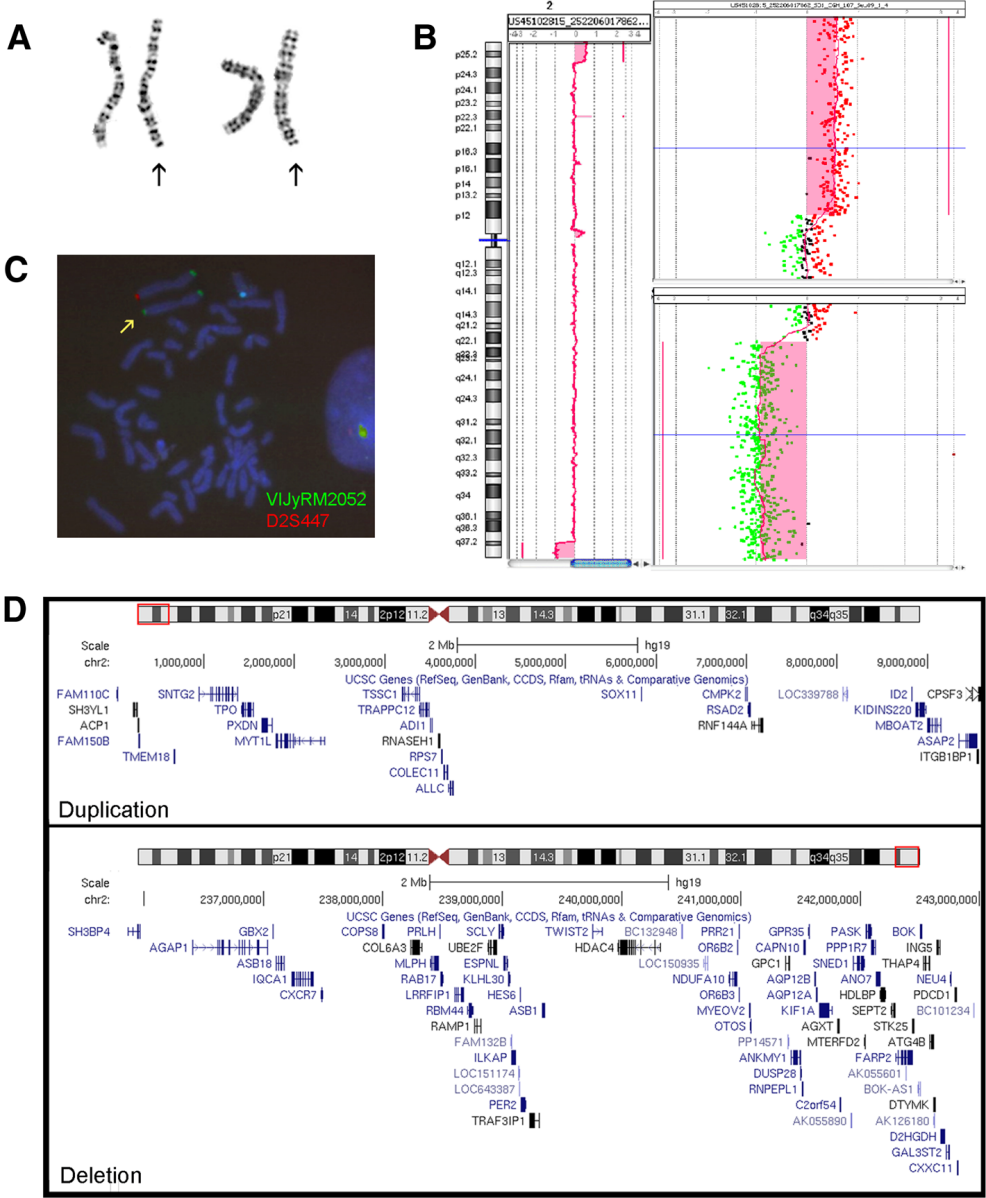
**Karyotype, array-CGH analysis, FISH analysis. A)** Cut-out of chromosomes 2 from two metaphases. The der(2) are highlighted by the arrows. **B)** Array-CGH profile of patient’s chromosome 2 showing the 2p distal duplication (upper panel in the enlargement) and the concurrent 2q distal deletion (lower panel in the enlargement). The experiment was performed by using a 180 K platform (180 K SurePrint G3 Human Kit, Agilent Technologies, Santa Clara, CA) as reported elsewhere [8]. **C)** FISH analysis with subtelomeric chromosome 2 specific probes (VIJyRM2052 in green, 2p; D2S447, in red, 2q; (Vysis TelVysion, Abbott Molecular, Abbott Park, IL) shows green signals at both extremities of the rearranged chromosome 2 (yellow arrow), flanked by its normal homolog. **D)** The protein-coding genes included in the duplication (upper panel) and the deletion (lower panel) are reported, according to UCSC Genome Browser (http://genome-euro.ucsc.edu/).

Because of facial dysmorphisms, mitral valve dysplasia, growth failure and mild developmental delay, Noonan syndrome was considered in the differential diagnosis. However, the sequencing of the *PTPN11* gene, the PTPN11 gene, whose mutations can explain about 50% of cases of Noonan syndrome [8], did not disclose any alteration. Since the prenatal karyotype resulted to be normal, a high-resolution banding karyotype was requested, and an abnormality at the distal long arm of one chromosome 2 was observed (Figure 4A). To characterize the rearrangement, array-CGH analysis was performed by using a 180 K platform (Agilent Technologies, Santa Clara, CA) [9]. A call for copy number variation (CNV) was done by at least three consecutive probes with a log_2_ratio value significantly different from 0, by using Genomic Workbench v. 5.0.14 software (Agilent, ADM-2 algorithm with a threshold of 5). This analysis showed a complex genomic imbalance consisting of a 2p25 distal duplication of about 9.6 Mb and a 2q37 distal deletion of about 7.3 Mb, containing 26 and 61 protein coding genes respectively: arr[hg19] 2p25.3p25.1(30,341-9,588,369)x3,2q37.2q37.3(235,744,424-243,041,305)x1 (Figure 4B-D). Fluorescent In Situ Hybridization (FISH) analysis, performed with subtelomeric chromosome 2 specific probes (VIJyRM2052 for 2p; D2S447 for 2q, both from Vysis TelVysion, Abbott Molecular, Abbott Park, IL) confirmed the presence of the two imbalances and showed that the duplicated 2p region was located distally at 2 q (Figure 4C). Since the rearrangement was detectably by high-resolution banding, the same karyotype analysis was performed on parental blood samples showing that the rearrangement was *de novo*.

## Conclusion

Both 2p duplications and 2q deletions are usually observed as part of more complex duplication/deletion syndromes, being distal imbalances frequently associated with the unbalanced segregation of a reciprocal translocation present in one parent or, more rarely, arisen de novo in the early embryo. Obviously, the presence of two different imbalances, involving several genes at once, makes clear genotype-phenotype correlations more difficult and renders the recognition of the syndrome on clinical grounds almost impossible. Complex rearrangements involving a terminal short arm duplication and a terminal long arm deletion of the same chromosome may result from meiotic recombination of a parental pericentric inversion as in one patient reported by Armstrong et al., 2005 [6]. However many of these cases are the result of a de novo event [10], suggesting that the rearrangement may occur in two steps, the first resulting in a terminal deletion lately repaired by the capture of the telomere of the opposite arm, thus resulting in its duplication [9,11]. In our case both parents had a normal karyotype, suggesting that the rearrangement most likely occurred through this mechanism.

In the literature only three viable patients with both a short arm duplication and long arm deletion of chromosome 2 have been reported [6,7], two of them sharing with our patient short stature, mild trigonocephaly, ptosis, low set ears, micrognathia, fleshy fingertips, scoliosis and abnormal hearing [6]. In the third case, in which the extent of 2p duplication was not assessed, developmental delay, short stature and dolichocephaly were reported [7]. Phenotypic findings in patients with chromosome 2q terminal deletions correlate in some cases with breakpoints positions [2,12], although in many cases genotype–phenotype correlations have been impaired by the lack of molecular analysis or by the presence of a translocation derivative. However, almost all patients have moderate to severe developmental delay, short stature, obesity and brachymetaphalangy [2,12,13]. The latter, together with short stature, is characteristic of the Albright’s hereditary osteodystrophy-like (AHO-like) syndrome in patients with 2q37 deletions. Haploinsufficiency for the *HDAC4* (Histone deacetylase 4) gene has been proposed as responsible for the observed brachymetaphalangy and intellectual disability, with both deletions and truncating mutations reported in association to this phenotype [3]. Although short stature seems to be a common trait for both 2q terminal deletions or 2p distal duplications, the presence of associated endocrinological defects has been rarely reported, and we are aware of only two reports of GH-deficiency in patients with a 2q37 deletion [13,14]. We were not able to find any cases of pituitary stalk interruption in patients with similar rearrangements, either searching the literature or in public databases such as DECIPHER [15]. In our patient 26 and 61 protein coding genes are respectively duplicated or deleted. Even using prioritization tools (Endeavour [16], ToppGene [17]), we were not able to find a convincing candidate for his pituitary defect. Moreover, no pituitary-specific expression is reported for any of the candidates. We can speculate that in the non-deleted chromosome, a gene necessary for the proper pituitary development is mutated, possibly unmasking a recessive condition.

In conclusion, this is the first case of distal 2p duplication and 2q deletion with severe short stature and pituitary hypoplasia, interrupted pituitary stalk and an ectopic posterior pituitary lobe. These findings are considered prognostic markers of permanent GH deficiency as detected in our patient and may be associated with other hormonal defects causing variably severe panhypopituitarism [18] not present in our case. The diagnosis of GH deficiency was confirmed by the significant increase in growth rate after substitutive GH therapy.

## Consent

Written informed consent was obtained from the patient’s parents for the publication of this report and any accompanying images.

## Abbreviations

GH: Growth hormone; MRI: Magnetic Resonance Imaging; CGH: Comparative Genomic Hybridization; FISH: Fluorescent In Situ Hybridization; IGF-I: Insulin-like Growth factor I; SD: Standard deviation score.

## Competing interests

The authors declare that they have no competing interests.

## Authors’ contributions

AV: she made substantial contribution to acquisition, analysis and interpretation of data; she also revised the manuscript critically; SP: she made substantial contribution to acquisition of data and she has been involved in drafting the manuscript; MS: she revised the manuscript critically and gave final approval of the version to be published; MS: she made substantial contribution to interpretation of data and revised the manuscript critically; EB: she has been involved in drafting the manuscript; CM: she has been involved in drafting the manuscript and revised the manuscript critically; OZ: she made substantial contribution to conception design and interpretation of data; she revised the manuscript critically and gave final approval of the version to be published; MB: he made substantial contribution to conception design and gave final approval of the version to be published. All authors read and approved the final manuscript.
